# Compression of the Umbilical Cord during Forceps Delivery

**Published:** 1893-12

**Authors:** Joseph Griffiths Swayne

**Affiliations:** Professor of Midwifery in University College, Bristol, and Consulting Physician-Accoucheur to the Bristol General Hospital


### COMPRESSION OF THE UMBILICAL CORD DURING FORCEPS DELIVERY.

BY

JOSEPH GRIFFITHS SWAYNE, M.D. Lond.,

*Professor of Midwifery in University College, Bristol, and Consulting Physician-Accoucheur to the Bristol General Hospital.*

---

FIFTEEN years ago I read a paper on "The Effects of Forceps Delivery on the Infant" at the annual meeting of the British Medical Association, which took place in Bath in 1878. During the previous year Dr. Galabin had published in the *Obstetrical Journal* for December, 1877, a paper on "The Effects of a Frequent and Early Use of the Midwifery Forceps upon the Fœtal and Maternal Mortality." After bringing forward a mass of statistics which bore upon this subject, Dr. Galabin arrived at the conclusion that "it has not been shown that the majority, or any considerable proportion, of the still-births which now occur in Britain would be preventable by a more timely resort to forceps." In my own paper I arrived at a similar conclusion to this; and I brought forward in illustration several cases I had witnessed of injuries received during forceps delivery which had in some instances been fatal to the child, and in others had produced rather serious, but fortunately not permanent, after-effects. Upon the subject to which I am now directing attention, I said: "The next four still-births out of the twenty-three are due to a cause of which I do not remember to have seen any notice by others: I mean *compression of the umbilical cord by the forceps*. In one of these the forehead presented to the left acetabulum. There were no particular signs of undue pressure on the head; but I observed when the child was born that the end of one blade of the forceps was compressing the cord (which happened to be round the child's neck) against the angle of the jaw. In another, the head had been much elongated by pressure; but I attributed death in this case also to the cord having been nipped between the edge of one blade and the jaw. In another, the same thing had happened, the head being but little altered in shape. In the fourth, the contour of the head was but little altered; but a loop of cord which had prolapsed, though not sufficiently to be previously detected, was found, as soon as the head was delivered, to be between the posterior blade of the forceps and the left frontal bone. Here then we have a source of danger to the child, arising simply from the use of the forceps, and which cannot be guarded against by any ordinary amount of skill. Nothing is more common than coiling of the cord round the child's neck, and it is a condition which cannot be recognised by ordinary vaginal examination before applying the forceps. If, therefore, one blade of the forceps should pass just an inch too far beyond the ear, it would be very likely to cause such an accident, especially if the head be so high that the ear cannot be felt, or present unfavourably, for instance with the forehead in the anterior semi-circle of the pelvis, or the head be much altered in shape and elongated by protracted labour." [1]

Up to that time I had met with 153 cases of forceps delivery in difficult labour; in 23 of these the child was either born dead or died shortly afterwards, making a death-rate of about 1 in $6\frac{2}{3}$. In four instances, as I had then recorded, death was caused by compression of the umbilical cord by the forceps, so

[1] *Brit. Med. Journ.*, 1878, vol. ii., p. 459.

that this peculiar cause of still-birth (which, as far as I know, had not then been recognised by British obstetric authorities) occurred to me as often as once in 38 cases and a fraction. There could be no doubt about it; for the birth of the child's head with the forceps *in situ* at once revealed this instrument as the direct cause of the infanticide. In order to ascertain whether my experience of forceps cases up to 1878 is confirmed by those I have recorded from that time up to the present, I may state that since 1878 I have attended 71 forceps cases with a death-rate of 12 infants, or, I should rather say, 10; for in one case (one of hydramnios) extensive desquamation of the cuticle showed that the child had been dead for several days; in the other, the child died soon after its birth from a very large exomphalos, accompanied with ectopia cordis. Thus we have 10 still-births in 71 cases, which is at the rate of 1 in $7\frac{1}{10}$ cases, or less than the death-rate of 1 in $6\frac{2}{3}$ recorded before 1878. Amongst the 10 still-births there were 2 in which death was clearly due to pressure of the forceps on the cord, which was coiled round its neck—once in one case and twice in the other. On comparing the two lists of cases—viz., those before 1878 and those which occurred subsequently up to the present time,—I find that the latter show a more favourable infant death-rate from forceps delivery than the former; for in the former it was at the rate of 1 in $6\frac{2}{3}$ cases, and in the latter of 1 in $7\frac{1}{10}$, both of which are better than 17 per cent. which Dr. Spiegelberg estimates to be the ordinary fœtal mortality from forceps delivery. In the first list of 153 cases, the death of the infant was in four instances clearly due to pressure by the forceps on the umbilical cord, and in the second list of 71 cases in two instances; the frequency of infant death from this cause being in the former at the rate of 1 death in 38 and a fraction, and in the latter of 1 in $35\frac{1}{2}$. The result of my statistics is, that in a total of 224 forceps cases I have noted the deaths of 6 infants from cord pressure—*i.e.* 1 in $37\frac{1}{2}$ cases; and the general result of my forceps experience since 1878 is, that I see no reason to alter the opinion which I expressed at that time.

The reading of my paper at Bath was followed by some discussion. In the course of it Dr. McClintock, the president in the Obstetrical Section, made the general remark that he considered that "more children had been saved by the early use of the forceps than destroyed." He did not, however, bring forward any facts or statistics in proof of this view. The same may be said of the remarks which fell from other speakers. The fact is, that just at that time the forceps was in high favour in every part of the United Kingdom, and especially in Dublin, in consequence of the excellent results as regards the mother, which had lately followed its more frequent and early use. Owing to this feeling accoucheurs were then, I think, too much disposed to take a *couleur de rose* view of the effects of this instrument, particularly upon the child. Although the much-increased and more skilful use of the long forceps, especially in England of late years, has led to the saving the lives of infants that would formerly have been destroyed by craniotomy, yet this is, I think, barely a set-off against the death of 1 infant in 37 cases which I have shown by my own experience to be the result of unavoidable pressure by the forceps on the umbilical cord, and which, of course, increases *pari passu* with the more frequent use of the forceps. In 1878 I was astonished to find no notice of this cause of infantile death in the standard obstetric works; and since then it has not, I think, received the attention its importance deserves. For instance, in Spiegelberg's excellent text-book of Midwifery, which was translated and published by the New Sydenham Society in 1888, the only reference I can find to this accident occurs in the following passage: "The compression of the skull which accompanies even the easiest forceps operations is apt to be fatal to the child, quite apart from the risk of the point of the blade causing death by *pressing on the cord which is coiled round the neck*, or on the larynx or cervical blood-vessels of the child. These facts explain how it is that the mortality of the children is comparatively high (17 per cent.), even with the simplest forceps operations."[1] With this last remark I entirely agree, and also with Dr. Galabin's conclusion quoted at the beginning of this paper.

[1] Vol. ii., p. 566.

